# Supplementary figures and images for: Rapid Indentification of Auramine O Dyeing Adulteration in Dendrobium officinale, Saffron and Curcuma by SERS Raman Spectroscopy Combined with SSA-BP Neural Networks Model
Source: Foods. 2023 Nov 14;12(22):4124. doi: 10.3390/foods12224124 (PMC10670709; doi:10.3390/foods12224124)

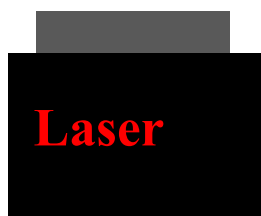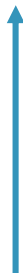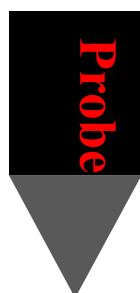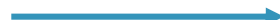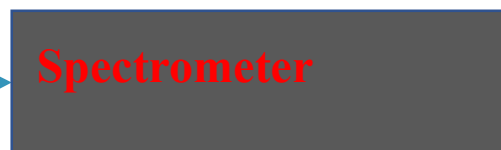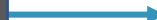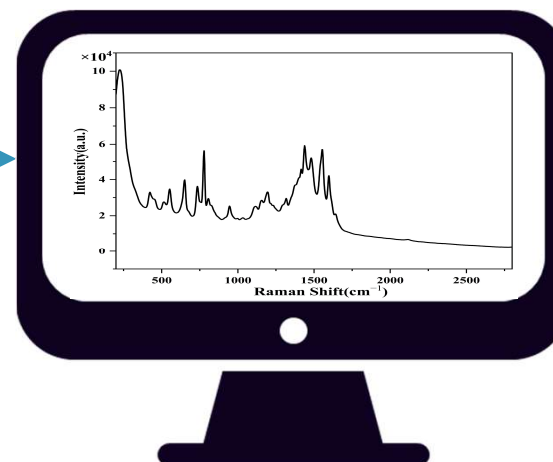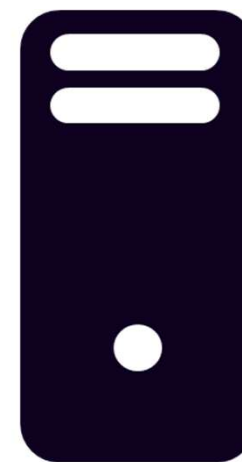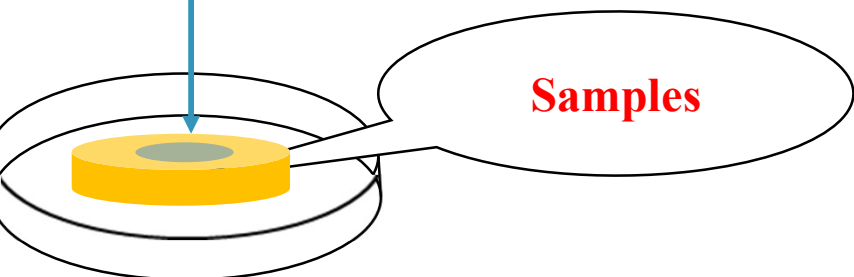

Supplement: Supplementary file 1 [file foods-12-04124-s001.zip › Figure S1.pdf]
